# Macromolecule Orientation in Nanofibers

**DOI:** 10.3390/nano8110918

**Published:** 2018-11-07

**Authors:** Dan Tian, Chun-Hui He, Ji-Huan He

**Affiliations:** 1 National Engineering Laboratory for Modern Silk, College of Textile and Engineering, Soochow University, 199 Ren-ai Road, Suzhou 215123, China; dtian@suda.stu.edu.cn; 2 Department of Chemistry, Xi’an-Jiaotong Liverpool University, Suzhou 215123, China; mathew_he@yahoo.com

**Keywords:** macromolecule, laminar flow, electrospinning, hierarchical structure

## Abstract

Electrospinning is now commercially used for the fabrication of nano/micro fibers. Compared with spider dragline silk, artificial fibers have poor mechanical properties. Unlike natural silk, which has a hierarchical structure with an approximate 3-fold symmetry, the molecular structure of spun fiber has neither folding nor orientation. To date, it is almost impossible to control molecule orientation during the spinning process. Here, we show that macromolecule orientation can be easily controlled using the laminar flow of fluid mechanics. A lasting laminar flow in a long needle can order macromolecules. We find that the orientation of macromolecules can greatly affect the morphology and mechanical properties of fibers. We expect our technology to be helpful for more sophisticated fabrication of fibers with ordered macromolecules and DNA-like twists.

## 1. Introduction

Hierarchical structures in nature always behave extremely well, with astonishing functional properties that no manmade materials can match. For examples, the hierarchical cocoon imposes no barricade to oxygen diffusion or water vapor [1], the hierarchical structure of a gecko’s foot enables the animal to adhere any smooth surface and detach easily at will [2,3,4], the hierarchical lotus leaf provides super-hydrophobicity [5,6,7]. If a hierarchy begins at the molecular level, the material requires far less building materials than it would beginning with a higher level. In this view, natural hierarchical structures are optimal in achieving a needed property; math-biomimicking was suggested in reference [8] in the design of an artificial hierarchy with the same fractal dimension as those of the natural one. Natural hierarchies always serve as a source of inspiration for scientists who hope to fabricate self-assembled macromolecular-scale nano/micro fibers embodying special functions, such as those of spider silk [9,10]. Nature always uses the simplest material blocks, which has rather meager properties, to design multi-scale materials with outstanding bio-functional properties, providing scientists with infinite inspiration to fabricate bio-inspired hierarchical structural materials using the simplest possible material blocks to achieve the highest performance. Molecular movement in some molecular devices can be effectively controlled by chemical methods like molecular rotary motors [11], synchrotron radiation-induced techniques [12], and molecular motors [13]. A mechanical technique using the simplest possible laminar flow has never been used before. Electrospinning is widely used to prepare various materials for specific applications [14,15,16], and Richard-Lacroix and Pellerin have made important contributions to molecular orientation in electrospun fibers. They showed the importance of controlling molecular orientation for some new and promising applications of molecule-oriented nanofibers [17] in mechanical engineering, thermal science, electrical devices, optical equipment, and puncture-proof fabrics [18,19,20,21,22]. In fluid dynamics, the laminar flow indicates that when a spun solution goes through a needle, the flow velocity varies gradually from zero on the needle’s inner-surface to the maximum along the cross-sectional center of the needle [23,24,25]. This velocity distribution is helpful to make macromolecules ordered. In this paper, we will use laminar flow theory to control the molecular movement in the jet of the process, which might be used for the fabrication of the finest hierarchical layer in future; thus, it would be possible to arrange macromolecules in a controllable way, like that of DNA structures. No report exists so far on controlling macromolecule orientation in nanofibers prepared by electrospinning. In this paper, we choose long needles in the electrospinning process to order macromolecules by laminar flow. X-ray diffraction (XRD), scanning electron microscopy (SEM), a mechanical property testing machine, and transmission electron microscopy (TEM) are used to reveal that the molecular movement can be effectively controlled by the needle’s length; a longer needle organizes macromolecules in a better order.

## 2. Materials and Methods 

### 2.1. Materials 

Polyvinyl alcohol (PVA) particles (Aladdin Industrial Corporation, Shanghai, China) were used in our experiment without any further purification; the sample was stored at room temperature, and its alcoholysis degree was 97.5–99.0%. 

Titanium dioxide (TiO_2_) nanoparticles (Shanghai Macklin Biochemical Co., Ltd., Shanghai, China) served as an additive, and were used as received. 

### 2.2. Instrumentation 

The electrospun nanofiber morphology was analyzed using a S4800 cold field scanning electron microscope (SEM, Hitachi S-4800, Tokyo, Japan). The experimental operation followed the manufacturer’s guidelines. To determine the diameter distribution of nanofibers, 500 fibers from 50 SEM images were chosen for diameter distribution analysis using ImageJ software (National Institute of Mental Health, Bethesda, MD, USA).

Tensile test was measured by the mechanical property testing machine (INSTRON-3365, INSTRON Company, Norwood, MA, USA). The fiber membrane was sheared as a rectangle with a width of 2 cm and a length of 4 cm, as required. The thickness of the fiber membrane was measured using a micrometer. All experimental data given in this paper were the averages of at least 3 measurements. In the test, the holding length was 20 mm and the tensile speed was 20 mm/min. Each sample was tested five times.

Semi-blunt puncture properties were measured by the mechanical property testing machine (INSTRON-3365, INSTRON Company, Norwood, MA, USA). The fiber membrane was sheared as a square membrane with a width of 10 cm, as required. The thickness of the fiber membrane was measured by a micrometer. All experimental data given in this paper were the averages of at least 5 measurements. In the test, the drop speed of the penetrator was 10 mm/min. Each sample was tested five times.

The pore size distribution was measured for the nanofiber membranes by capillary flow porometry (Porometer 3G, Quan-tachrome Instruments, Boynton Beach, FL, USA). As required, all samples were circular membranes with diameters of 25 mm. Porofil wetting solution was used in our experiment. 

The X-ray diffraction (XRD) patterns were analyzed by X-ray diffractometer/X ‘Pert-Pro MPD (PANalytica, Philips Company, Amsterdam, Netherlands), and the Peakfit software was used for peak fitting of XRD patterns to determine the crystalline components and amorphous components for estimation of the crystallinity.

Transmission electron microscopy (TEM) of PVA nanofibers was obtained by transmission electron microscopy (Tecnai G2 F20 S-Twin, Hillsboro, OR, USA) operating at 200 kV. The operation process followed the manufacturer’s guidelines; the voltage was 200 kV, the dark current and the emission current were, respectively, 10.57 µA and 64 µA.

The resistance of the nanofiber membrane was measured by a high resistance meter (ZC36, Shanghai sixth electricity meter factory Co., Ltd., Shanghai, China). As required, all samples were circular membranes with diameters of 4.5 cm.

### 2.3. Solution Preparation

First, 1.6 g Polyvinyl alcohol (PVA) powder was put into 18.4 g deionized water with a temperature of 80 °C, and the mixture was then magnetically stirred on a heating magnetic stirrer (DF-101S, Xinrui Instrument Inc., Changzhou, China) until a transparent solution was obtained. 

### 2.4. Electrospinning Process

The electrospinning process was similar to that of our previous publications [26,27,28]. In our experiment, all spinning conditions were kept unchanged except the needle’s length, which was 6 mm, 11 mm, 16 mm, 21 mm, 26 mm, 38 mm, 60 mm, 100 mm, and 150 mm. The obtained PVA solution was put into a syringe, which was mounted in a syringe pump (JZB-1800, JYM instrument Inc., Shanghai, China). A high voltage power supply (DW-P303-1ACF0, High Voltage Electronics Co., Tianjin, China) was used in our experiment. The voltage was 18 kV, the flow ratio was 1 mL/h, the distance between the needle and the collector was 20 cm, the temperature was 24 °C, and the relative humidity was 37%.

## 3. Theoretical Analysis

**Figure 1:**
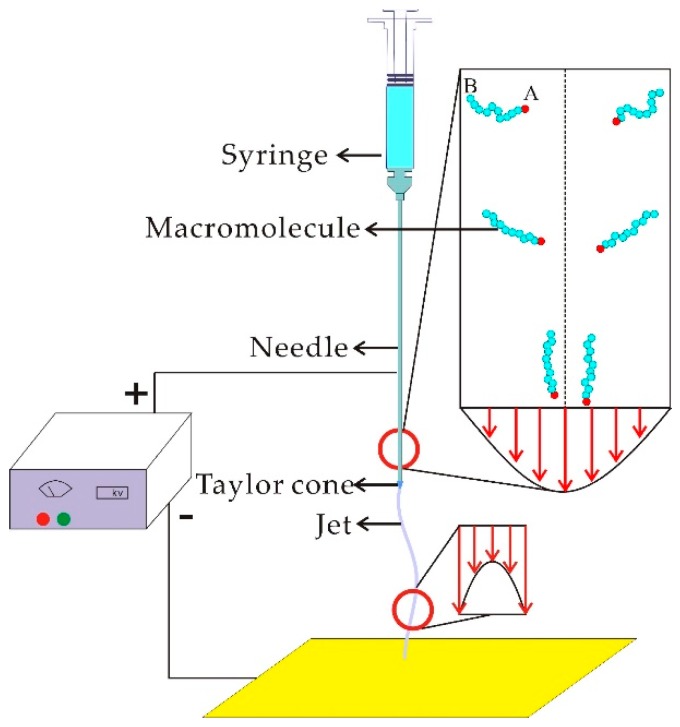
Electrospinning spinning controlled by the laminar flow in the long needle. The macromolecules in the needle will be gradually stretched and moved to the center along the line of flow (streamline); in contrast, the macromolecules in the jet move gradually outwards.

The experimental setup is shown in Figure 1, where the needle’s length is controlled while other parameters are fixed. When the spun solution enters into the needle, all macromolecules move in a random way, and might be entangled or distorted. Figure 1 also reveals the velocity distribution inside the needle. On the boundary, the flow velocity is zero, and on the boundary layer, where the viscous force dominates, the flow velocity increases gradually, reaching a maximum at the center. This velocity distribution is helpful to order the macromolecules. 

A macromolecule near the end of the solid surface moves slowly, while one near the center moves much more rapidly. As a result, the macromolecule is gradually stretched. On the other hand, following the Bernoulli principle:

(1) $$\frac{1}{2}u^{2}+\frac{P}{\rho }=B\;$$

where *u* is the flow velocity, *P* is the pressure, *ρ* is the density, and *B* is the Bernoulli constant; the macromolecule will gradually move into a line of flow (steamline). 

We label the two ends of the macromolecule as *A* and *B*; end *A* is near the center, while end *B* is close to the boundary. This means that:

(2) $$u_{A}>u_{B}\;$$

According to Equation (1), we have

(3) $$P_{B}-P_{A}=\frac{1}{2}\rho (u_{A}^{2}-u_{B}^{2})>0\;$$

This pressure difference can gradually push the end of *B* into the center, and finally, the stretched macromolecule is arranged along the steamline. A longer needle should produce more ordered macromolecules.

## 4. Discussion

### 4.1. Transmission Electron Microscopy

**Figure 2:**
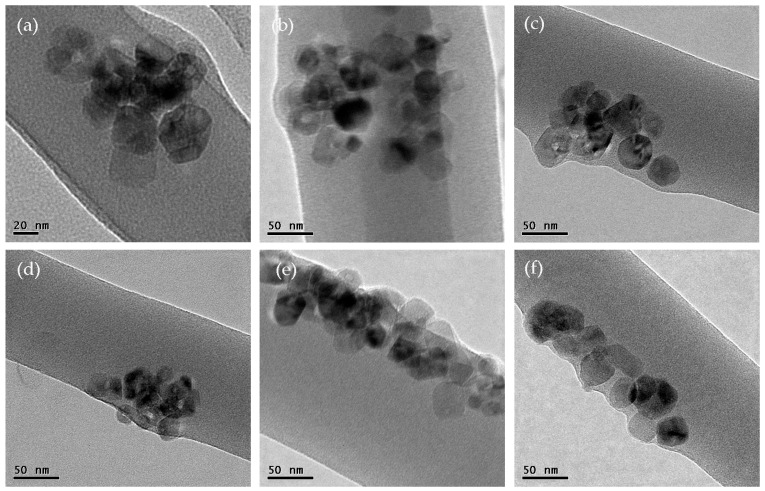
TEM images of PVA/TiO_2_ nanofibers prepared by different needles (**a**) 6 mm, (**b**) 16 mm, (**c**) 26 mm, (**d**) 60 mm, (**e**) 100 mm and (**f**) 150 mm.

**Figure 3:**
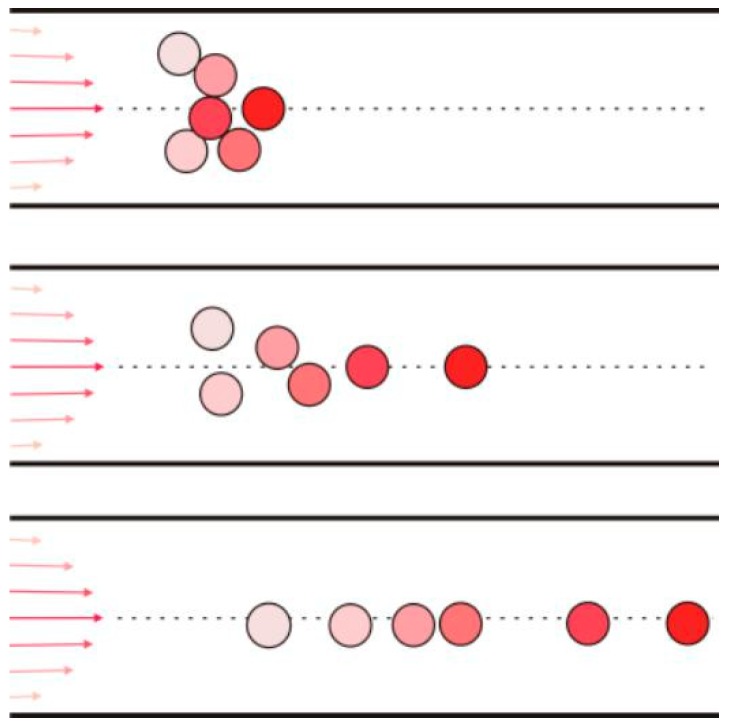
Nanoparticle movement in a long needle. The particle in the center moves fastest, and that far from the center moves slowly and is pushed gradually into the center.

In order to give visual verification of this theoretical analysis, we fabricated PVA/T_i_O_2_ nanofibers by electrospinning. In our experiment, titanium dioxide (T_i_O_2_) nanoparticles are used as additives to see their distribution in the nanofibers. The titanium dioxide nanoparticles are uniformly distributed in the spun solution. In our experiment, 0.3 g titanium dioxide nanoparticles were dispersed in 20 g of PVA solution using a high speed homogenizing apparatus (FA25-JX, Fluko Company, Berlin, Germany) for 30 min. The nanoparticles are easy to aggregate together in the solution, and the aggregated T_i_O_2_ nanoparticles can be observed inside the PVA/T_i_O_2_ nanofibers when the needle is short enough, as shown in Figure 2a,b. When the needle’s length increases, the aggregated particles will gradually become more ordered, as shown in Figure 2c–f. A longer needle results in a more ordered streamline. Figure 3 gives an illustration of the nanoparticle movement through a long needle.

During the spinning process, the electrostatic force acts only on the surface of the moving jet, so the jet surface has a higher velocity than that at the center; the velocity distribution on the section of the jet is illustrated in Figure 1. According to the Bernoulli equation given in Equation (1), the nanoparticles will be pushed gradually from the center to the boundary, as shown in Figure 2f. The TEM illustrations give us a good visual verification of our theoretical prediction: a longer needle results in a more ordered streamline.

### 4.2. XRD Spectra

**Figure 4:**
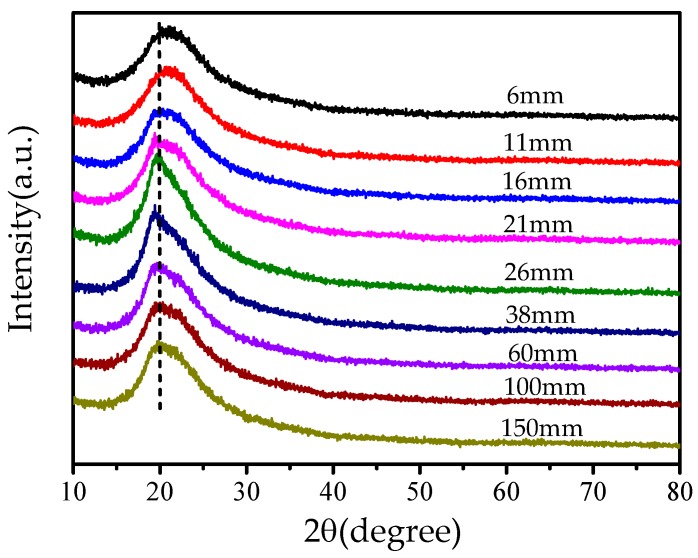
X-ray diffraction (XRD) spectra of PVA nanofiber membranes prepared by different needles.

In order to verify the macromolecule orientation in the fiber, we used XRD spectra to measure the crystallinity of the obtained fibers. Crystallinity is a good index for the degree of structural order, and polymer molecule orientation greatly affects the crystallization of the polymer. Higher crystallization implies less chain entanglement [29]. X-ray diffraction (XRD) has been widely used to study crystal structure to reveal a molecule’s orientation in a polymer. In this paper, XRD analysis was applied to elucidate the effect of the needle’s length on the crystalline structure of PVA membranes; their XRD patterns with characteristic crystalline peaks are shown in Figure 4.

From Figure 4, it can be seen that a strong peak is located at around 2*θ* = 20° for PVA; this is the characteristic peak for an orthorhombic lattice (101). Normally, PVA should have two peaks at (101) and (200), but if the crystal is not strong enough, the two peaks will merge into one [30]. The sharp band given in Figure 4 corresponds to the region where crystals are well ordered, while the defused band implies an amorphous region. We use the Peakfit software to calculate the crystallinity of each sample using the following formula [31]

(4) $$\theta =\frac{A_{c}}{A_{c}+A_{a}}\;$$

where *A_c_* is the area of crystalline component, *A_a_* is amorphous component, the values of *A_c_* and *A_a_* can be determined by curve-fitting Gaussian technique with the Peakfit software. The strong signal at 19.8° was assigned to the crystalline component, and the broad signal at 19.6° was assigned to the amorphous component [29]. The crystallinity of PVA membranes obtained by different needles is listed in Table 1.

Like in the above analysis, the crystallinity is controllable by the laminar flow; a longer needle results in higher crystallinity. For an infinite length of needle, a fully-developed laminar flow is formed, and the molecule’s orientation is in an ideal periodic pattern. Additionally, its crystallinity reaches a maximum. On the other hand, when the needle’s length tends to zero, the flow is assumed to be in an amorphous state where macromolecules moves randomly and are entangled with each other. 

**Figure 5:**
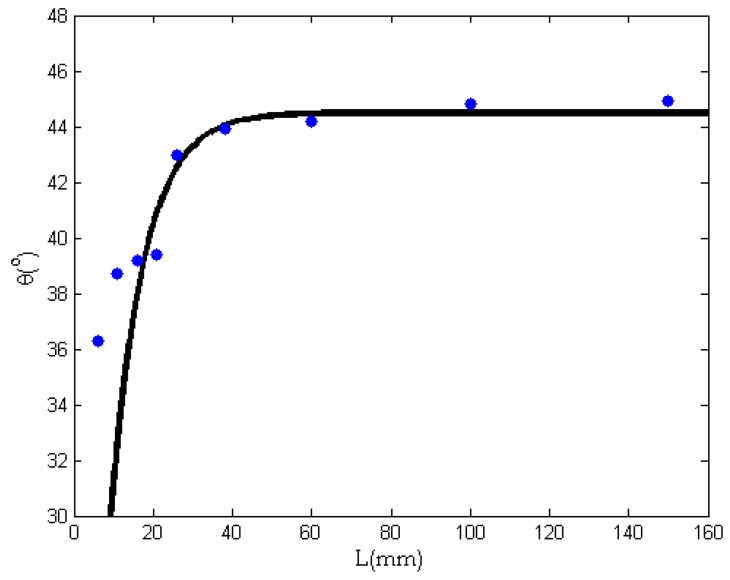
The relationship between needle length and crystallinity. Dots are experimental data; the continuous line is the theoretical prediction.

We assume that the crystallinity can be expressed as

(5) $$\theta =\theta_{0}(1-e^{-k_{1}L})\;$$

where *θ*_0_ is the maximal crystallinity, *L* is the needle’s length, and *k*_1_ is a constant. The parameters *θ*_0_ and *k*_1_ can be determined experimentally. Figure 5 is the experimental data showing the relationship between the crystallinity and the needle’s length. Using these experimental data, Equation (5) becomes

(6) $$\theta =44.5(1-e^{-0.12L})\;$$

The crystallinity increases exponentially from the minimum for the completely amorphous case to a final value of 44.5% (see Figure 5), where we can see that the experimental data and theoretical curve are in good agreement. The theoretical curve shows that the crystallinity of the fiber increases exponentially with an increase of the needle’s length. When the length reaches a threshold of about 50 mm, the crystallinity tends to be stable. This is because the crystallinity can be controlled by the laminar flow in the needle. When a fully developed laminar flow is established, the needle’s length will not affect the crystallinity very much. 

### 4.3. Morphological Characterization 

**Figure 6:**
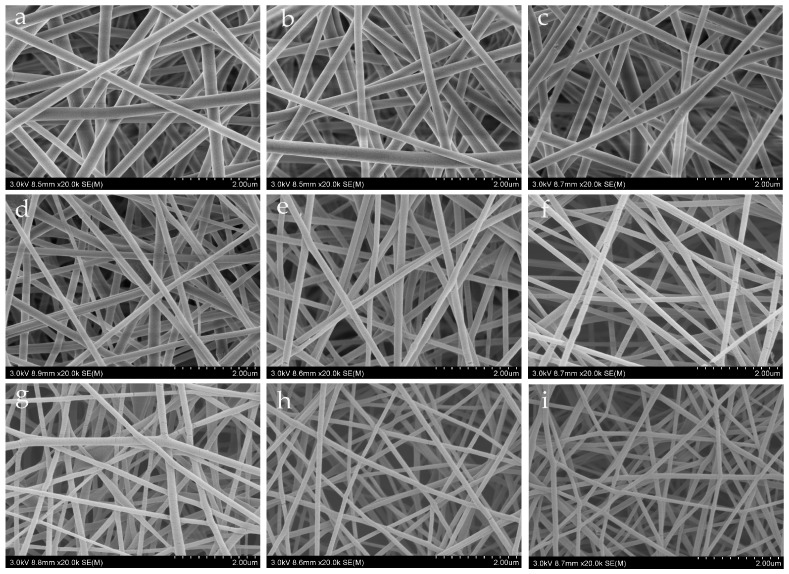
SEM images of PVA membranes obtained by different needles, (**a**) 6 mm, (**b**) 11 mm (**c**) 16 mm, (**d**) 21 mm, (**e**) 26 mm, (**f**) 38 mm, (**g**) 60 mm, (**h**) 100 mm, and (**i**) 150 mm.

SEM illustrations for different needles are given in Figure 6. It can be seen that the fiber diameter is greatly affected by the needle’s length. The average diameters of nanofibers obtained by different needles are listed in Table 2.

The moving jet can be considered as a steady one-dimensional flow. Mass conservation requires that

(7) $$\pi r^{2}\rho u=Q\;$$

where *r* is the radius of the jet, and *Q* is the mass flow rate. 

The momentum balance equation for a viscous fluid is written as

(8) $$\frac{d}{dz}(\frac{1}{2}u^{2})=-\frac{1}{\rho }\frac{d\tau }{dz}\;$$

where *τ* is the viscous force.

The integration of Equation (8) results in

(9) $$\frac{1}{2}u^{2}+\frac{\tau }{\rho }=B\;$$

Equation (9) reveals that higher viscous force leads to lower velocity. The viscous force depends upon crystallinity. As a simple analysis, we assume that the viscous force can be expressed in the form:

(10) $$\tau =\tau_{0}(1-k_{2}L)\;$$

where *τ*_0_ is the viscous force for the amorphous condition, and *k*_2_ is a constant.

By the mass equation, Equation (7), we have

(11) $$r={\left(\frac{Q}{\pi \rho u}\right)}^{1/2}={\left[\frac{Q}{\pi \rho {\left(2B-2\tau /\rho \right)}^{1/2}}\right]}^{1/2}=r_{0}{\left(1+k_{3}L\right)}^{-1/4}\;$$

where $r={\left[\frac{Q}{\pi \rho^{1/2}{\left(2B\rho -2\tau_{0}\right)}^{1/2}}\right]}^{1/2}$, $k_{3}=\frac{2\tau_{0}k_{2}}{2B\rho -2\tau_{0}}$.

Figure 6 gives the experimental relationship between the fiber diameter and the needle length. After identification of the constants in Equation (11) by the experimental data, we have

(12) $$d=350{\left(1+0.5L\right)}^{-1/4}\;$$

**Figure 7:**
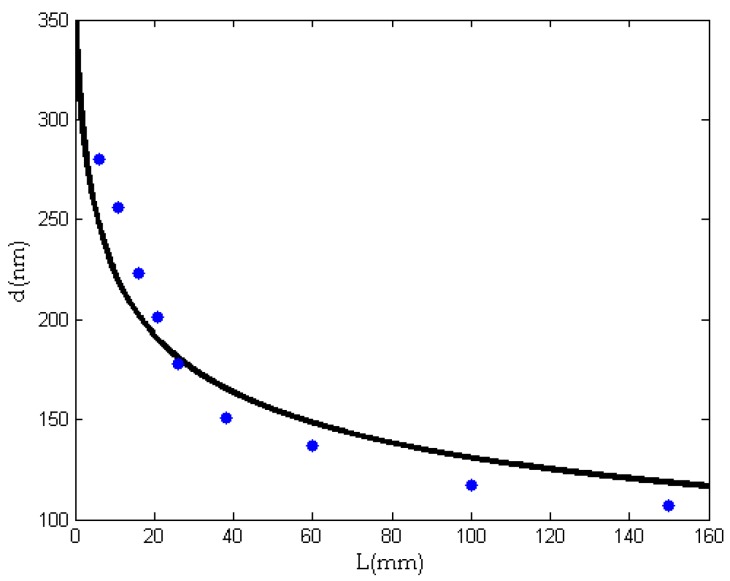
The effect of the needle’s length on the average diameter of nanofibers. Dots: experimental data; Continuous line: theoretical prediction.

The semi-empirical formula shown in Equation (12) describes an exponential fall from 350 nm, when the needle’s length is zero, to 107 nm, when *L* = 150 mm; see Figure 7. It can be seen from Figure 7 that the average diameter of the fiber decreases gradually with an increase of needle length, and the curve decreases rapidly when *L* is small. When the needle’s length increases to a critical value, the average diameter of the fibers becomes stable. 

### 4.4. Mechanical Property

Macromolecule orientation or crystallinity will greatly affect the mechanical properties. When a force is applied to the fiber, it has to overcome the friction among molecule chains, which might be entangled with each other. We use a modification of the capstan friction equation to express the friction force on molecule chains:

(13) $$f=f_{0}+\mu (e^{-k_{4}L}-1)\;$$

where *f* is the friction among chains, *f*_0_ is the friction when a macromolecule’s orientation is not controlled (*L* = 0), and *k*_4_ is a parameter relative to the entangled angle. When L→∞, all molecule chains are parallel; such a case yiels the highest crystallinity and lowest friction. 

The tensile strength can be expressed as

(14) $$\sigma =\sigma_{0}+f=\sigma_{0}+f_{0}+\mu (e^{-k_{4}L}-1)\;$$

where *σ*_0_ is stress when no molecule friction exists. 

**Figure 8:**
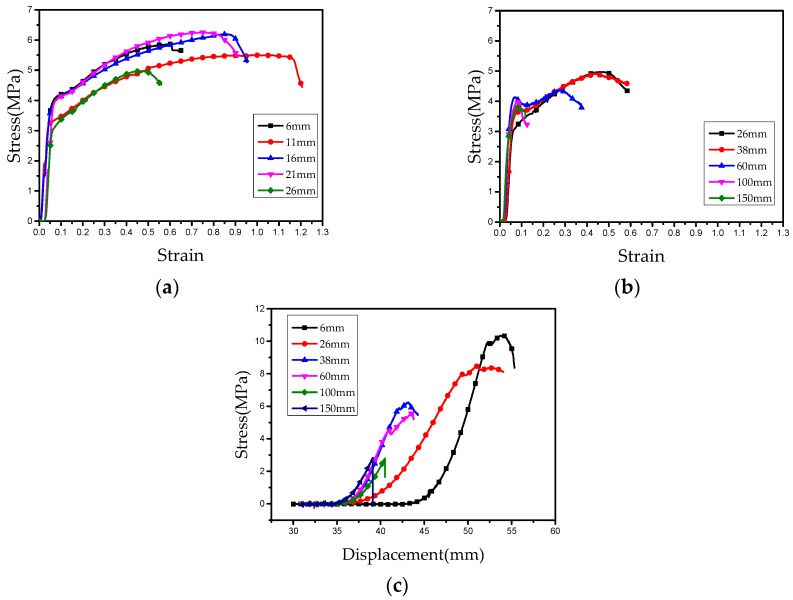
Mechanical property tests for tensile experiment (**a**,**b**); and for semi-blunt puncture test (**c**).

In order to verify Equation (14), we carried out a tensile experiment and a semi-blunt puncture test. The experimental results are given in Figure 8. It can be seen that the needle’s length greatly affects the mechanical properties. Using the experimental data, we can determine the constants in Equation (14) for the tensile experiment and the semi-blunt puncture experiment.

Tensile experiment:

(15) $$\sigma_{t}=10+6.3(e^{-0.05L}-1)\;$$

Semi-blunt puncture test:

(16) $$\sigma_{p}=10+9.8(e^{-0.01L}-1)\;$$

**Figure 9:**
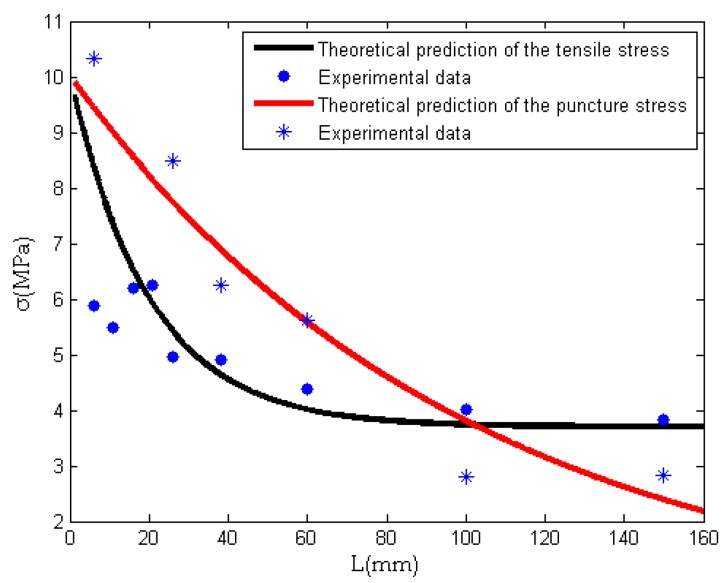
The relationship between needle length and mechanical properties.

Our theoretical predictions given in Equations (15) and (16) give a good trend of maximal tensile strength when the needle’s length increases; see Figure 9. The data of maximal tensile stress and maximal puncture stress of nanofiber membranes obtained by different needles are listed in Table 3 and Table 4. It can be seen from Figure 9 that the tensile stress and puncture stress of the fiber membrane decreases with an increase of needle length. The curve of tensile stress decreases rapidly at the beginning, and finally it tends to be gentle, while the curve of puncture stress keeps decreasing constantly. This is because with the increase of needle length, the macromolecules are more and more orderly in the moving jet, so the friction among molecule chains is smaller and smaller. The tensile stress decreases with the increase of needle length. When the order of macromolecules reaches the limit, the tensile stress will tend to a stable value. But when the puncture force acts on the fiber membrane, it not only overcomes the friction among molecule chains, but also cuts off some molecule chains, so when the tensile stress tends to a stable value, the puncture stress continues to decrease.

A spider silk, which consists of thousands of nanofibers, has excellent mechanical properties due to a very low *k*_4_ value. 

### 4.5. Surface Resistance 

Crystallinity will also affect electrical conductivity. Many experiments have found that an increase of crystallinity can result in huge conductivity enhancement [32,33,34]. Table 5 gives the experimental data for different lengths of needle. 

In Table 5, *RS/L* is the resistivity of the nanofiber membrane, *R* is the resistance of the nanofiber membrane, *S* is the surface area of the nanofiber membrane to be measured, *L* is thickness of the nanofiber membrane, and *G* is the conductivity of nanofiber membrane. 

It is obvious that a longer needle produces a nanofiber with higher crystallinity, which leads to higher conductivity, as illustrated in Table 5. 

### 4.6. Gas Permeability

**Figure 10:**
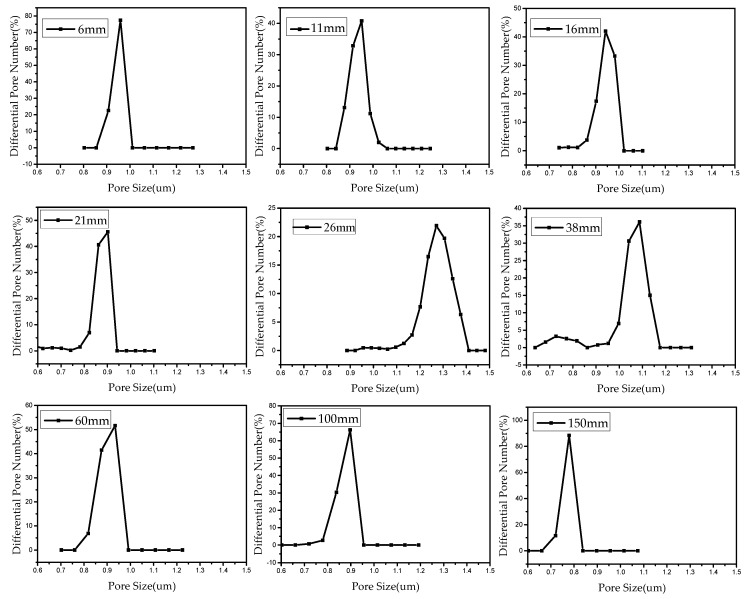
Pore size distributions of PVA membranes prepared by different needles.

Pore size has an important effect on the filtration properties of fiber membranes. The pore size distribution of PVA membranes was measured by a capillary flow porometry. Figure 10 shows the pore size of PVA membranes prepared by different needles.

Our previous study revealed that the average pore size is directly proportional to the fiber diameter [35].

(17) D∝d 

While the fiber diameter can be determined by the needle length, see Equation (12), so the pore size can be expressed in the form:

(18) $$D=k{\left(1+0.5L\right)}^{-1/4}\;$$

where *k* is a constant. Using the experimental data, the constant involved in Equation (18) can be determined, and Equation (18) becomes

(19) $$D=2.2{\left(1+0.5L\right)}^{-1/4}\;$$

**Figure 11:**
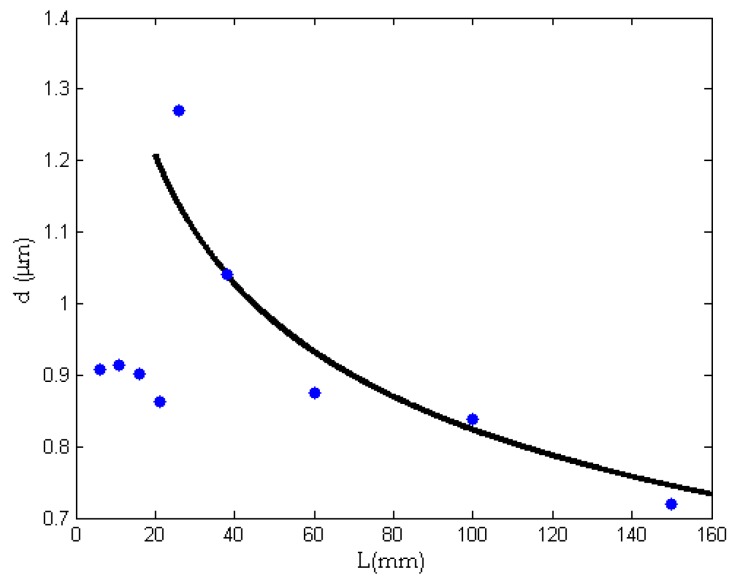
The relationship between needle length and pore size. Dots: experimental data; the continuous line: theoretical result by Equation (19).

Figure 11 illustrates the relationship between needle length and pore size. It can be seen from Figure 11 that beginning with a needle length of 26 mm, the pore size of the fiber membrane keeps decreasing with the increase in needle length. This is because the average diameter of the fibers decreases gradually with the increase of needle length. As discussed above, the pore size scales with the fiber diameter [35].

## 5. Conclusions

In this paper, for the first time, we observed that the laminar flow in a long needle can be used to control macromolecule orientation in a nanofiber, It was observed that a longer needle places macromolecules in a better order. Our theory analysis was cross-certified using various experimental data from images obtained using scanning electron microscopy (SEM) and transmission electron microscopy (TEM). The mechanical property testing machine was used to determine the mechanical properties of the nanofiber membranes. All events show that the laminar flow can effectively control macromolecule- or nanoparticle- distribution in nanofibers. The controlled macromolecules can be used as a first cascade of an artificial hierarchy, mimicking natural ones like spider silks. This paper suggests a promising method for fabricating artificial hierarchical materials at a molecular level. Our method might shed a light on both nanotechnology and bio-mimics.

## Figures and Tables

**Table 1:** The crystallinity of PVA membranes obtained by different needles.

Needle’s Length (mm)	Average Crystallinity (%)	Standard Deviation (σ) (%)	Confidence Interval (%)
6	36.3	0.239	0.21
11	38.7	0.343	0.30
16	39.2	0.238	0.21
21	39.4	0.356	0.31
26	43	0.158	0.14
38	43.9	0.336	0.29
60	44.2	0.230	0.20
100	44.8	0.207	0.18
150	44.9	0.238	0.21

**Table 2:** The relationship between needle length and the average diameter of nanofibers.

Needle’s Length (mm)	$\mathrm{Average}\;\mathrm{Diameter}\;(\bar{D})$ (nm)	Standard Deviation (σ) (nm)	Confidence Interval (nm)
6	280	42.2	±8.3
11	256	38.6	±7.6
16	223	32.8	±6.4
21	201	33.4	±6.6
26	178	21.8	±4.3
38	151	19.5	±3.8
60	137	19.3	±3.8
100	117	17.6	±3.4
150	107	17.3	±3.4

**Table 3:** The relationship between needle length and the maximal tensile stress of nanofiber membrane.

Needle’s Length (mm)	$\mathrm{Average}\;\mathrm{Stress}\;(\bar{D})$ (MPa)	Standard Deviation (σ) (MPa)	Confidence Interval (MPa)
6	5.87	0.12	±0.11
11	5.50	0.16	±0.14
16	6.20	0.08	±0.07
21	6.25	0.12	±0.11
26	4.97	0.11	±0.10
38	4.92	0.15	±0.13
60	4.38	0.11	±0.10
100	4.01	0.17	±0.15
150	3.84	0.15	±0.13

**Table 4:** The relationship between needle length and the maximal puncture stress of nanofiber membrane.

Needle’s Length (mm)	$\mathrm{Average}\;\mathrm{Stress}\;(\bar{D})$ (MPa)	Standard Deviation (σ) (MPa)	Confidence Interval (MPa)
6	10.34	0.20	±0.17
26	8.48	0.24	±0.21
38	6.24	0.18	±0.16
60	5.61	0.22	±0.19
100	2.79	0.17	±0.15
150	2.83	0.20	±0.17

**Table 5:** The relationship between needle length and the resistivity of nanofibers.

Needle Height (mm)	Resistance (*R*) (Ω)	Resistivity (*RS/L*) (Ω⋅m)	Conductivity (*L/RS*) (S·m^−1^)
6	2.2 × 10^12^	6.2 × 10^13^	1.61 × 10^−14^
26	3.1 × 10^12^	5.6 × 10^13^	1.79 × 10^−14^
150	1.1 × 10^12^	2.7 × 10^13^	3.7 × 10^−14^
